# Remarkable static and dynamic NLO response of alkali and superalkali doped macrocyclic [hexa-]thiophene complexes; a DFT approach†

**DOI:** 10.1039/d0ra08099c

**Published:** 2021-01-20

**Authors:** Hasnain Sajid, Faizan Ullah, Sidra Khan, Khurshid Ayub, Muhammad Arshad, Tariq Mahmood

**Affiliations:** Department of Chemistry, COMSATS University Islamabad Abbottabad Campus Abbottabad-22060 Pakistan mahmood@cuiatd.edu.pk; Institute of Chemistry, University of the Punjab Lahore Pakistan

## Abstract

In this study, the nonlinear optical (NLO) response of alkali metal atom (Li, Na and K) and their corresponding superalkali (Li_3_O, Na_3_O and K_3_O) doped six membered cyclic thiophene (6CT) has been explored. The optimized geometries of complexes; Li@6CT, Na@6CT, K@6CT, Li_3_O@6CT, Na_3_O@6CT and K_3_O@6CT depict that the superalkalis and alkali metals interact through the active cavity of 6CT. Interaction energies reveal that superalkalis have higher interaction with 6CT than alkali metals. The nonlinear optical (NLO) response of the reported complexes is estimated *via* both static and dynamic hyperpolarizabilities which are further rationalized by the HOMO–LUMO gap, natural bond orbital (NBO) charge transfer, dipole moment, polarizabilities and *β*_vec_. A remarkably high NLO response is computed for Na_3_O@6CT among all of the complexes. The static hyperpolarizability of the Na_3_O@6CT complex is 5 × 10^4^ au along with the highest *β*_vec_ value (2.5 × 10^4^ au). High charge transfer (1.53*e*^−^) and small *E*_H–L_ gap (2.96 eV) is responsible for such a large NLO response. For dynamic NLO responses, electro-optic Pockel's effect (EOPE) and second-harmonic generation (SHG) are explored. A very large quadratic nonlinear optical response (3.8 × 10^−12^ au) is observed for the Na_3_O@6CT complex. Moreover, the absorption spectrum of the Na_3_O@6CT complex shows ultra-high transparency in the ultraviolet and visible regions unlike any other of its counterparts.

## Abstract 2

In this study, the nonlinear optical (NLO) response of alkali metal atom (Li, Na and K) and their corresponding superalkali (Li_3_O, Na_3_O and K_3_O) doped six membered cyclic thiophene (6CT) has been explored.

## Graphical abstract

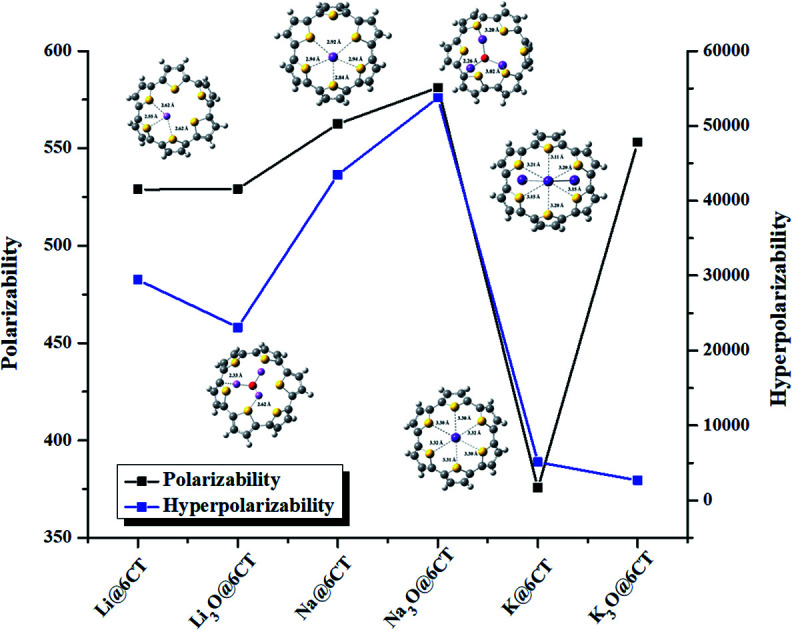

## 1 Introduction

To date, significant consideration has been devoted to the fabrication of high performance nonlinear optical (NLO) materials,^1–4^ due to their utility in electro-optic devices.^5–7^ To fulfil this demand, various kinds of novel NLO materials with excess electrons have been designed.^8,9^ It is very well documented that the excess electrons play a pivotal role in the large NLO response.^10,11^ Excess electrons can be generated by capping a material with alkali metal atoms.^12^ Thus alkali metal doping leads to large first and second hyperpolarizabilities of the systems.^13,14^ Munsif *et al.*,^15^ reported the exceptionally high NLO response of inorganic nanocages upon doping with alkali metal atoms. In their study, the authors claimed that the hyperpolarizability of potassium doped boron phosphide (inorganic nanocage) was the highest (7.9 × 10^5^ au) ever reported. Maria and co-workers illustrated the exceptional increase in the hyperpolarizability of inorganic systems to 1.3 × 10^4^ au from 0.00 au (pure material) upon doping with potassium metal.^16^ In another similar report, Maria *et al.*,^17^ studied the nonlinear optical behaviour of inorganic nanocages. Their results suggested the hyperpolarizability of such systems was greatly enhanced upon capping with alkali metal atoms. Like inorganic systems, the NLO behaviour of organic systems is equally enhanced on complexation with alkali metals. For example, Li *et al.*,^8^ theoretically designed a novel electride by adsorbing lithium metal on calix[4]pyrrole. The resulting electride molecule showed a large hyperpolarizability value (7326 au) which was twenty times larger than that of isolated calix[4]pyrrole systems. Based on the above discussion, it can be concluded that the doping of alkali metals remarkably enhance the opto-electronic properties of inorganic as well as organic systems.

In the recent decades, superalkalis, a kind of superatom clusters are attaining more interest as compared to alkali metal atoms. The superalkalis have greater affinity of donating valance electrons due to the lower vertical ionization potential.^18^ Therefore, their usage in nonlinear optics advocated significant interest of experimental and theoretical researchers. It has been observed that the superalkalis doping increases the hyperpolarizability of the inorganic materials such as aluminium phosphide,^19^ boron phosphide^20^ and silicon carbide nanocages.^21^ Apart from the inorganic systems, the NLO response of organic materials doped with superalkalis is also investigated. The organic molecules are expected to have giant NLO response because of their extended π-conjugation.^22–24^ In this regard, the nonlinear behaviour of graphdiyne was measured upon doping with superalkalis. Shehzadi *et al.*,^25^ reported the dramatic increase in the first hyperpolarizability of superalkalis doped graphdiyne to 7.7 × 10^4^ au. In another similar report, the remarkable enhancement in the NLO response of graphdiyne sheet upon complexation with superalkalis is demonstrated by Kosar *et al.*^26^ The authors compared the NLO response of M_3_O and M_3_S (M = Li, Na & K) complexed graphdiyne and observed that both showed equal effect. In our previous report, we have presented that superalkalis (M_3_O) doping in cyclic oligofuran remarkably enhances the NLO response. We reported that the cyclic conjugated ring systems can effectively be used as high NLO material due to their electron dense active cavity.^27^

The area of alkali and superalkalis doped cyclic thiophene ring system needs to be further explored. Thus, here we intend to compare the NLO response of macrocyclic hexathiophene ring (6CT) upon complexation with alkali metal atom (Li, Na, K) and their superalkalis analogues (Li_3_O, Na_3_O and K_3_O). The geometric, electronic and nonlinear optical properties of alkali and superalkalis doped 6CT *i.e.*, Li@6CT, Na@6CT, K@6CT, Li_3_O@6CT, Na_3_O@6CT and K_3_O@6CT complexes are studied computationally. The NLO behaviour of said complexes are investigated *via* first hyperpolarizability (*β*_o_), electro-optical Pockels effect (EOPE), second harmonic generation (SHG), and nonlinear refractive index (*n*_2_) calculations.

## 2 Computational methods

Geometry optimizations of complexes are performed with two high level Minnesota functionals including M05-2X and M06-2X along with 6-31G(d,p), 6-31+G(d,p) and 6-311+G(d,p) basis sets. All calculations are performed with Gaussian09 software.^28^ The interaction energies of complexes are defined by the expression below (eqn (1)).

1 *E*_int_ = *E*_complex_ − *E*_6CT_ − *E*_SA/AA_

where *E*_complex_, *E*_6CT_ and *E*_SA/AA_ are the energies of alkali atoms/superalkalis@6CT complex, cyclic hexathiophene and superalkalis/alkali atoms, respectively. Frontier molecular orbital (FMO) and NBO analysis are performed for the estimation of HOMO–LUMO (H–L) gap and amount of charge transfer of 6CT, respectively, upon complexation with alkali/superalkalis. To confirm the charge transfer and variation of H–L gaps, density of state (DOS) spectra are plotted through GaussSum software.^29^ The polarizability, first hyperpolarizability calculations are benchmarked at M05-2X, M06-2X and LC-BLYP functionals with 6-31+G(d,p) basis set. However, the dynamic NLO responses of complexes are calculated at LC-BLYP because it is reported as a best functional for the estimation of nonlinear optical properties due to the full range separation with 1.00 correct fraction of nonlocal exchange functional.^15,30^ The polarizability (*α*_o_), first hyperpolarizability (*β*_o_) and second order hyperpolarizability *β*_vec_ are defined as:2
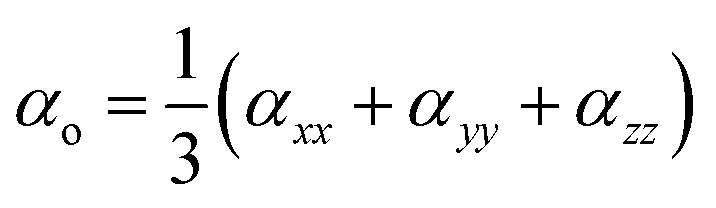

3 *β*_o_ = (*βx*^2^ + *βy*^2^ + *βz*^2^)^1/2^

4
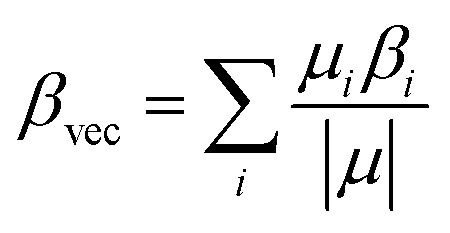
where *β*_*x*_ = *β*_*xxx*_ + *β*_*xyy*_ + *β*_*xzz*_, *β*_*y*_ = *β*_*yyy*_ + *β*_*xzz*_ + *β*_*yxx*_, *β*_*z*_ = *β*_*zzz*_ + *β*_*zxx*_ + *β*_*zyy*_

5 *β*_*i*_ = ∑(*β*_*ijj*_ + *β*_*jji*_ + *β*_*jij*_)*i*, *j* = {*x*, *y*, *z*}

The second hyperpolarizability (*γ*_o_) is estimated as:
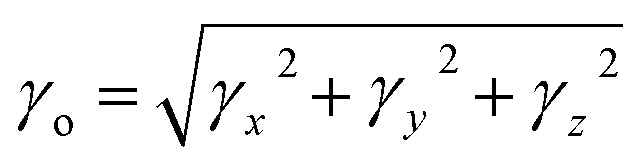


The frequency dependent NLO response in terms of the second harmonic generation (SHG) *β*(−2*ω*, *ω*, *ω*), the electro-optical Pockels effect (EOPE) *β*(−*ω*, *ω*, 0), electro-optic Kerr effect (EOKE) form *γ*(−*ω*, *ω*, 0, 0) and SHG form *γ*(−2*ω*, *ω*, *ω*, 0) are estimated at two standard Nd:Yag laser frequencies: *ω* = 0.0428 au (1064 nm) and *ω* = 0.0856 au (532 nm).

The frequency dependent/dynamic NLO response is computed through Coupled Perturbed Kohn–Sham (CPKS) method^31^ and results are analyzed through Multiwfn software.^32^ The dynamic first hyperpolarizability can be represented using the following equation:*β*(*ω*) = [*β*_*x*_^2^(*ω*) + *β*_*y*_^2^(*ω*) + *β*_*z*_^2^(*ω*)]^1/2^where the coefficients of second harmonic generation (SHG) are obtained using*β*_*i*_ = *β*_*iii*_(−2*ω*, *ω*, *ω*) + *β*_*ijj*_(−2*ω*, *ω*, *ω*) + *β*_*ikk*_(−2*ω*, *ω*, *ω*)

And the coefficients of electro-optical Pockels effect (EOPE) are obtained from*β*_*i*_ = *β*_*iii*_(−*ω*, *ω*, 0) + *β*_*ijj*_(−*ω*, *ω*, 0) + *β*_*ikk*_(−*ω*, *ω*, 0).

## 3 Results and discussion

### 3.1 Optimized geometries and stabilities

**Fig. 1:**
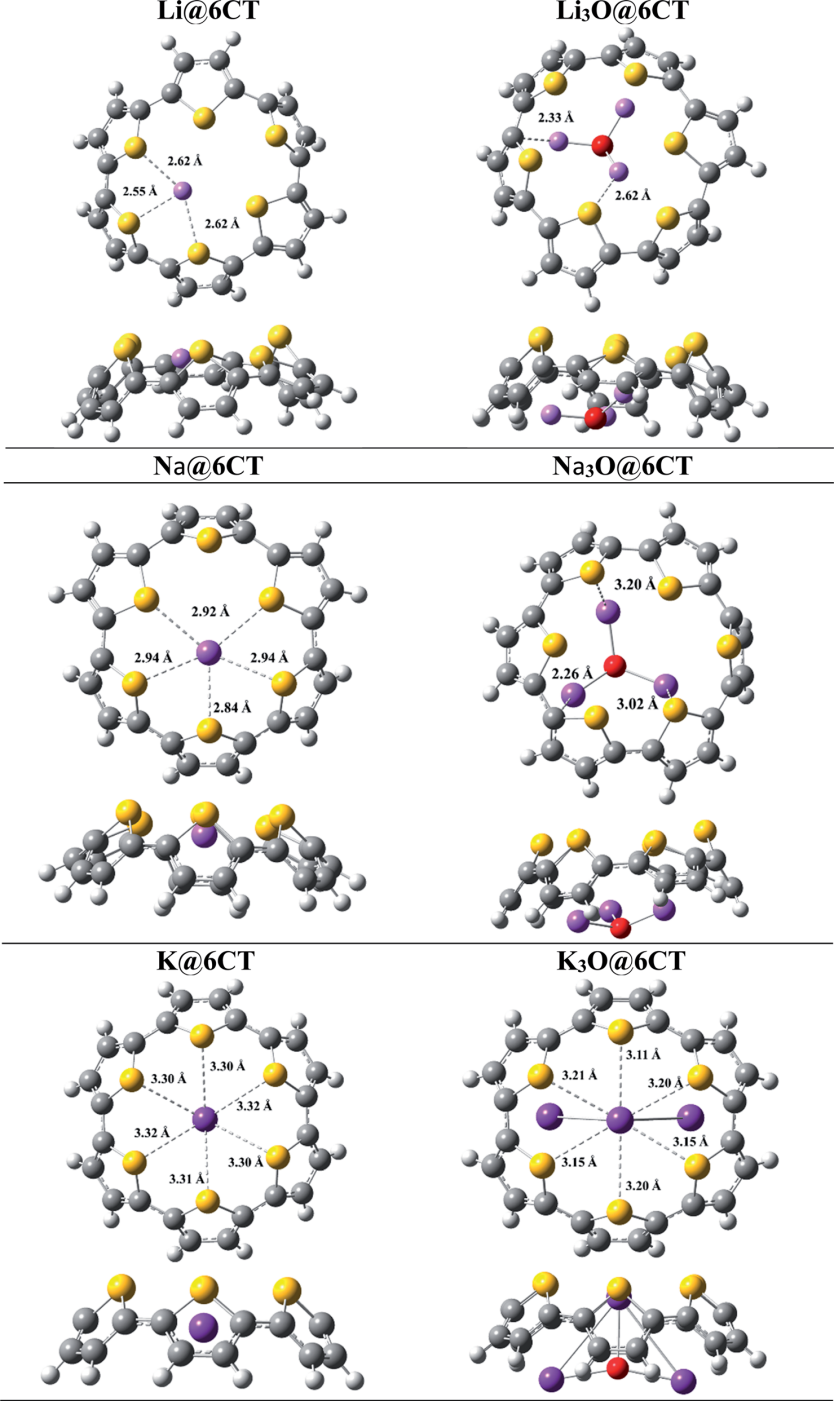
Top and side view of most stable optimized geometries of AA@6CT and SA@6CT complexes.

The six membered cyclic thiophene ring (6CT) is doped with three alkali metal atoms (Li, Na, K) and superalkalis (Li_3_O, Na_3_O and K_3_O) to form AA@6CT (alkali metal doped) and SA@6CT (superalkalis doped) complexes. The different orientations of interactions are possible due to the various sensitive positions of 6CT. All these orientations are studies and their geometries are given in ESI (Fig. S1–S3).† Among all possibilities, the most stable geometries are considered for detailed analysis and their structures are displayed in Fig. 1. The interaction energies of AA@6CT and SA@6CT complexes are calculated at two Minnesota functionals *i.e.*, M05-2X & M06-2X. The selection here is based on the previous reports, revealing the higher accuracy of Minnesota functionals to estimate the noncovalent interactions. For even better analysis, calculations are performed by using 6-31G (d,p), 6-31+G(d,p) and 6-311+G(d,p) basis sets. The interaction energies of complexes are listed in Table 1. Broadly, the interaction energies of SA@6CT complexes are higher at all methods as compared to AA@6CT complexes which might be due to the higher electron density on superalkalis than alkali atom. Among all the complexes, the highest interaction energies are computed for Li_3_O@6CT complex at M05-2X functional. The interaction energies of Li_3_O@6CT complex are −76.60, −71.86 and −70.34 kcal mol^−1^ at M05-2X/6-31G(d,p), M05-2X/6-31+G(d,p) and M05-2X/6-311+G(d,p) level of theory, respectively.

**Table 1:** Benchmark interaction energies (in kcal mol^−1^) of SA@6CT and AA@6CT complexes at different level of density functional theory

Complexes	M052X			M062X			
	6-31G (d,p)	6-31+G (d,p)	6-311+G (d,p)		6-31G (d,p)	6-31+G (d,p)	6-311+G (d,p)
Li@6CT	−41.07	−40.29	−41.90		−39.54	−38.85	−40.44
Li_3_O@6CT	−76.60	−71.86	−70.34		−72.76	−67.66	−67.37
Na@6CT	−35.59	−35.10	−36.58		−33.41	−32.94	−34.53
Na_3_O@6CT	−64.41	−59.67	−61.95		−60.90	−56.18	−58.54
K@6CT	−40.51	−39.83	−42.19		−39.34	−38.72	−41.11
K_3_O@6CT	−71.19	−61.45	−60.82		−73.76	−64.29	−66.06

Similarly, in case of AA@6CT complexes, lithium doped complex also shows higher stability with the interaction energies of −41.07, −40.26 and −41.90 kcal mol^−1^ at 6-31G (d,p), 6-31+G(d,p) and 6-311+G(d,p) basis sets, respectively. Quite similar to M05-2X, Li and Li_3_O doped complexes also exhibit higher stability at M06-2X with a small exception at 6-31G(d,p) basis set. The interaction energies of Li_3_O@6CT complexes are −72.76, −67.66 and −67.37 kcal mol^−1^ while Li@6CT complex are −39.54, −38.85 and −40.44 kcal mol^−1^ at M06-2X/6-31G(d,p), /6-31+G(d,p) and /6-311+G(d,p), respectively. Owing to the smallest atomic size, the lithium atom interacts more closely to the 6CT which is further evident by the least interaction distance (*I*_d_) among all SA@6CT and AA@6CT complexes. The interaction distances of Li_3_O@6CT and Li@6CT complexes are 2.62 and 2.55 Å, respectively (see Table 2) which are least among all the other complexes.

**Table 2:** Interaction parameters of optimized geometries of SA@6CT and AA@6CT complexes

Complexes	Sym	Int bond	Int distance (Å)	HOMO (eV)	LUMO (eV)	*E*_H–L_ (eV)	*Q* (*e*^−^)
6CT	*C*_1_	—	—	−6.43	−0.24	5.02	—
Li@6CT	*C*_1_	Li⋯S	2.55	−4.58	−1.31	3.27	0.60
Li_3_O@6CT	*C*_1_	Li⋯S	2.62	−4.59	−1.44	3.15	1.69
Na@6CT	*C*_1_	Na⋯S	2.84	−4.54	−1.00	3.14	0.76
Na_3_O@6CT	*C*_1_	Na⋯S	3.02	−4.50	−1.54	2.96	1.53
K@6CT	*C*_1_	K⋯S	3.30	−4.01	−1.48	2.93	0.75
K_3_O@6CT	*C*_1_	K⋯S	3.11	−4.54	−1.24	3.29	0.91

The stability of the lithium-based complexes is followed by the potassium doped complexes. The high stability of K_3_O@6CT complex can be explained by examining the geometry. Fig. 1 reveals that only a single K atom equally interacts with all the sulfur atoms of 6CT unlike Na doped complexes where interaction of sodium is with some sulfur atoms. The interaction distances (K⋯S) of K or K_3_O and 6CT are 3.30 and 3.11 Å, respectively. The average interaction distances between sodium atom of Na and Na_3_O with sulfur atoms of 6CT are 2.84 and 3.02 Å, respectively. The interaction energies of K_3_O@6CT and K@6CT complexes are higher at all methods than Na_3_O@6CT and Na@6CT complexes, respectively (see Table 1). It can be seen from the results, the basis set 6-31G(d,p) (without polarization) overestimates the energy values while 6-31+G(d,p) basis set predicts higher accuracy in the results.

### 3.2 Electronic properties

**Fig. 2:**
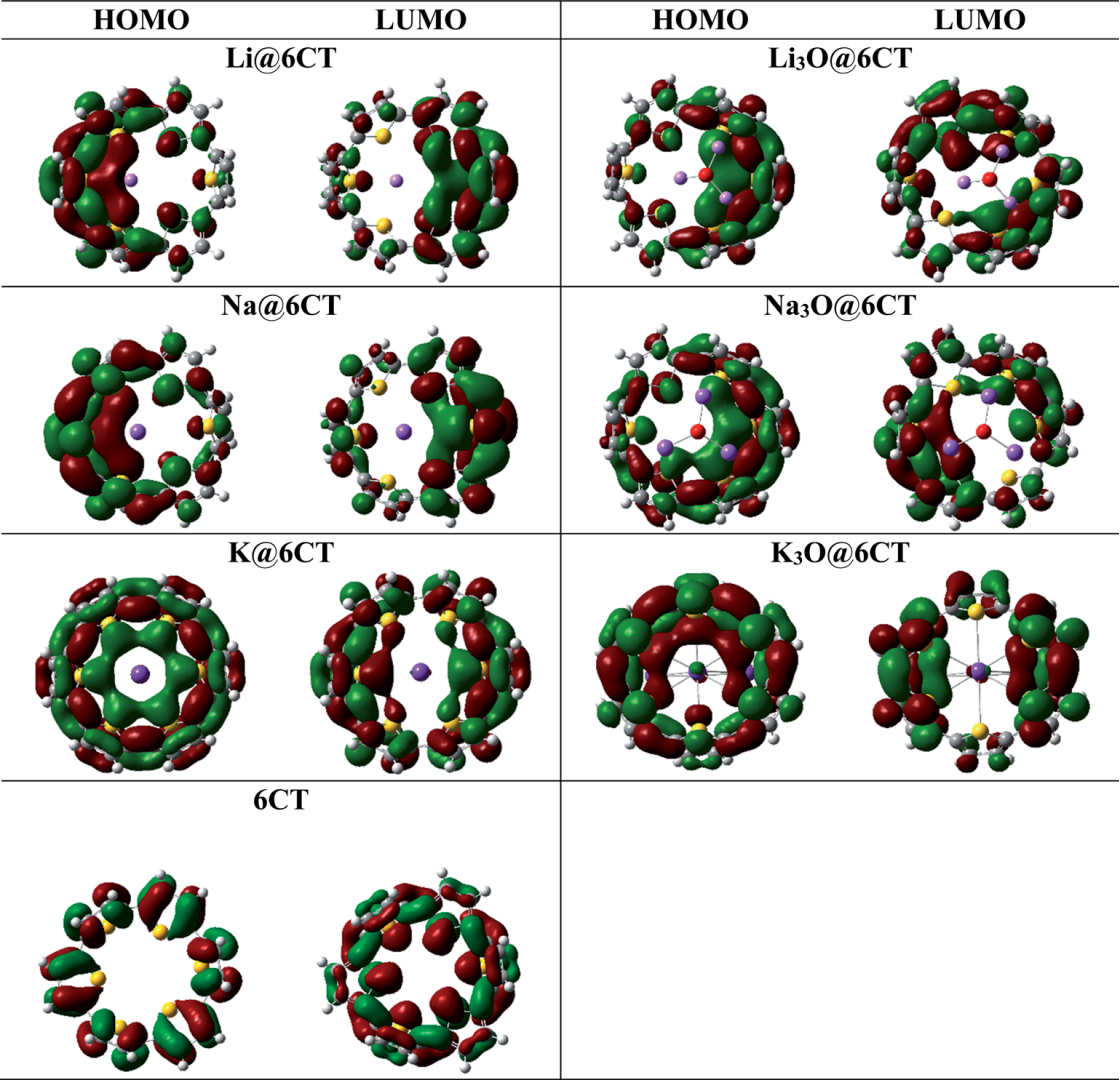
HOMO and LUMO orbitals of 6CT, AA@6CT and SA@6CT complexes.

**Fig. 3:**
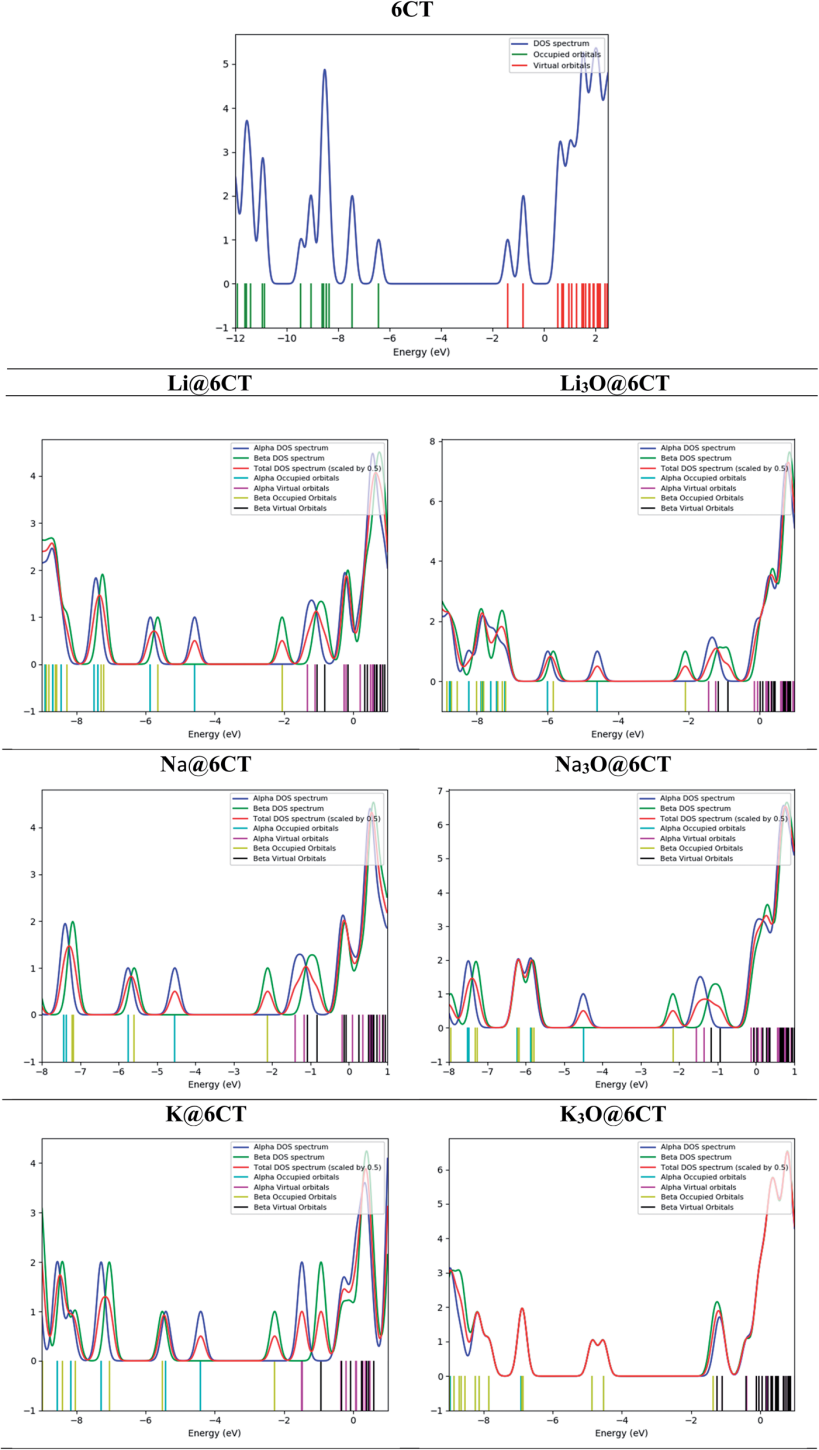
DOS spectra of 6CT, AA@6CT and SA@6CT complexes.

Molecular orbital analysis is performed to examine and compare the variation of electronic parameters of 6CT upon complexation with alkali atoms and superalkalis. The energies of HOMO and LUMO orbitals and their differences (*E*_H–L_) in eV are given in Table 2. The E_H-L_ of isolated 6CT is 5.02 eV where the energies of HOMO and LUMO are −6.43 and −0.24 eV, respectively. The *E*_H–L_ of 6CT decreases upon complexation due to the formation of new energy levels.^33^ In comparison with alkali metals, the superalkalis doping widely reduces the H–L gaps which reflects the large availability of excess electrons in SA@6CT complexes. The *E*_H–L_ of 6CT is reduced to 3.15, 2.96 and 3.29 eV in Li_3_O@6CT, Na_3_O@6CT and K_3_O@6CT complexes, respectively. On the other hand, the *E*_H–L_ of Li@6CT, Na@6CT and K@6CT complexes are 3.27, 3.14 and 2.93 eV, respectively. The decrease in the H–L gaps of complexes is due to the variation in the energies of HOMO and LUMO orbitals upon complexation with alkali metals and superalkalis. For instant, the LUMO energy of 6CT (−0.24 eV) reduces to −1.31, −1.00 and −1.48 eV on interaction with Li, Na, and K atoms, respectively. Similarly, the LUMO energies of Li_3_O@6CT, Na_3_O@6CT and K_3_O@6CT complexes are shifted to −1.44, −1.54 and −1.24 eV, respectively. The decreasing trends of LUMO energies of AA@6CT and SA@6CT complexes are as follow; K > Li > Na and Na_3_O > Li_3_O > K_3_O, respectively. Moreover, the trend of increasing energies of HOMO orbital for AA@6CT and SA@6CT complexes are; K > Na > Li and Na_3_O > K_3_O > Li_3_O, respectively. The effects of K and Na_3_O in H–L gaps of AA@6CT and SA@6CT complexes, respectively are more pronounced. Therefore, the K@6CT and Na_3_O@6CT complexes show the lowest H–L gap among in their series. Furthermore, DOS spectra reveal (Fig. 3) that the generation of new HOMO/occupied energy levels on interaction plays pivotal role to lower the *E*_H–L_ gaps of complexes. It is clearly seen in Fig. 2 that the HOMO orbitals of the systems mainly reside near the doped alkali atoms or superalkalis, indicating the generation of new HOMO orbitals which are further responsible of producing excess electrons in the system. The DOS spectrum in Fig. 3 reveals that the occupied and virtual orbitals of isolated 6CT are located at −6.43 and −0.24 eV, respectively. Upon complexation with alkali atoms and superalkalis, the spin state has been changed from singlet (in 6CT) to doublet (in AA@6CT and SA@6Ct complexes) due to the involvement of unpaired electrons from doped metal atoms. Moreover, the occupied orbitals of the 6CT are shifted closer to the Fermi level on interaction. The occupied orbitals appear at −4.58, −4.54, −4.01 eV (light blue colour) in Li@6CT, Na@6CT and K@6CT complexes, respectively. However, the occupied orbitals peaks of Li_3_O@6CT, Na_3_O@6CT and K_3_O@6CT complexes appear at −4.59, −4.50 and −4.54 eV, respectively. Moreover, the peaks (red colour) in the DOS spectrum of 6CT are also shifted toward Fermi level upon complexation with alkali and superalkalis (light brown peaks). From DOS analysis, it can be concluded that the HOMO and LUMO energy gaps of AA@6CT and SA@6CT complexes decrease due to the shift or generation of new orbitals close to the Fermi level.

NBO analysis is performed to examine the charge transfer between 6CT and AA or SA. Expectedly, the charge transfer in SA@6CT complexes is higher than AA@6CT complexes due to the large electronic density on superalkalis. The NBO charge transferred (*Q*) in Li_3_O@6CT, Na_3_O@6CT and K_3_O@6CT complexes is 1.69, 1.53 and 0.91*e*^−^, respectively. Probably, some of the electronic density is shifted from alkali atoms toward the covalently bonded electronegative oxygen atom of superalkalis. This charge transfer increases with decreasing the electronegativity of alkali atoms from Li to Na. Due to the increasing charge transfer between the atoms of superalkalis, the charge transfer decreases between superalkalis and 6CT from Li_3_O to Na_3_O. On the other side, the amount of charge transfer increases upon increasing atomic number of alkali atoms. The amounts of charge transfer is 0.60, 0.76 and 0.75*e*^−^ in Li@6CT, Na@6CT and K@6CT complexes, respectively.

### 3.3 UV-vis analysis

**Fig. 4:**
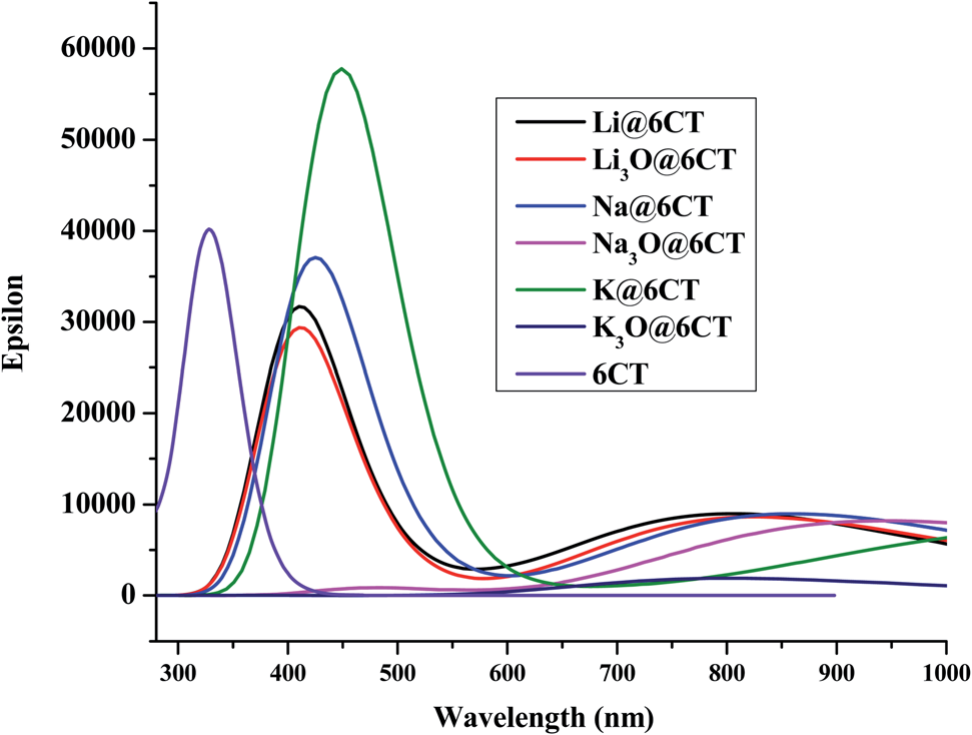
UV-vis spectra of 6CT, AA@6CT and SA@6CT complexes.

It is postulated that NLO active material should be transparent in the interested region of EMR spectrum.^34^ In order to find the adsorption wavelength of 6CT before and after doping, the UV-vis analysis is performed at TD/M05-2X/6-31+G(d,p). It is cleared from the results (Table 3 & Fig. 4) that the maximum absorption of isolated 6CT appears at 328 nm. The absorption spectrum of 6CT splits into two red shifted peaks in visible region upon complexation especially with Li, Na, K and Li_3_O. Firstly, the red shift in the UV-vis spectra of AA@6CT and SA@6CT complexes articulates the increasing π–π* transitions. Results reveal that all the reported materials are transparent in ultraviolet region. However, the Na_3_O@6CT and K_3_O@6CT complexes show good transparency in both ultraviolet and visible regions due to the absence of peak maxima between 200 to 800 nm. In comparison, Na_3_O@6CT and K_3_O@6CT complexes indicate adequate transparency for laser applications in both UV and vis regions.

**Table 3:** NLO parameters: dipole moment (*μ*_o_), polarizability (*α*_o_), first hyperpolarizability (*β*_o_), projection of *β* on dipole moment vector (*β*_vec_), hyper-Rayleigh (*β*_HRS_), second hyperpolarizability (*γ*_o_), crucial excitation energies (*E*_ex_), change in dipole moment (Δ*μ*), vertical ionization energy (*V*_IE_), maximum absorbance (*λ*_max_) and oscillator strength (*f*_o_) of isolated 6CT and AA@6CT & SA@6CT complexes

LC-BLYP											
Complexes units	*μ*_o_ D	*α*_o_ au	*β*_o_ au	*β*_vec_ au	*β*_HRS_ au	*γ*_o_ au	Δ*E*_ex_ eV	Δ*μ* D	*V*_IE_ eV	*λ*_max_ nm	*f*_o_ au
6CT	4.85	384.56	2 × 10^2^	—	—	—	0.85	1.19	6.96	328	0.49
Li@6CT	6.00	528.90	3 × 10^4^	1.5 × 10^4^	1.5 × 10^4^	2.0 × 10^6^	3.10	1.76	5.02	424	0.38
Li_3_O@6CT	6.50	529.20	2 × 10^4^	9.8 × 10^3^	1.1 × 10^4^	1.7 × 10^6^	3.10	1.38	5.03	425	0.28
Na@6CT	6.93	562.53	4 × 10^4^	2.3 × 10^4^	2.4 × 10^4^	4.1 × 10^6^	3.06	2.31	4.98	443	0.21
Na_3_O@6CT	6.89	581.13	5 × 10^4^	2.5 × 10^4^	2.7 × 10^4^	2.6 × 10^6^	1.66	6.19	4.94	883	0.09
K@6CT	7.27	375.62	5 × 10^3^	3.9 × 10^3^	2.3 × 10^3^	5.6 × 10^6^	2.96	1.34	4.92	448	0.71
K_3_O@6CT	13.17	553.15	3 × 10^3^	2.5 × 10^3^	1.3 × 10^3^	3.6 × 10^5^	2.39	2.95	6.33	809	0.04

## 4 Nonlinear optical properties

### 4.1 Static NLO responses

The values of dipole moment (*μ*_o_) and polarizabilities (*α*_o_) of isolated and complexed 6CT are given in Table 3. The dipole moment of 6CT is 4.85 D due to its *C*_1_ symmetry (Table 2). However, the dipole moment of 6CT increases upon complexation with alkali metals and superalkalis due to the distortion of symmetry. The dipole moments are ranging from 6.00 to 7.27 D and 6.50 to 13.17 D in AA@6CT and SA@6CT series, respectively. Similarly, the static polarizabilities of SA@6CT complexes are higher than AA@6CT complexes due to the greater electronic contribution of superalkalis. The polarizabilities of Na@6CT and Na_3_O@6CT complexes are the highest among all complexes which is well consistent with the hyperpolarizabilities (*vide infra*). The *α*_o_ of AA@6CT complexes is ranging from 375.62 au (K@6CT) to 562.53 au (Na@6CT) whereas, these values range from 528.90 to 553.15 au for SA@6CT complexes.

**Fig. 5:**
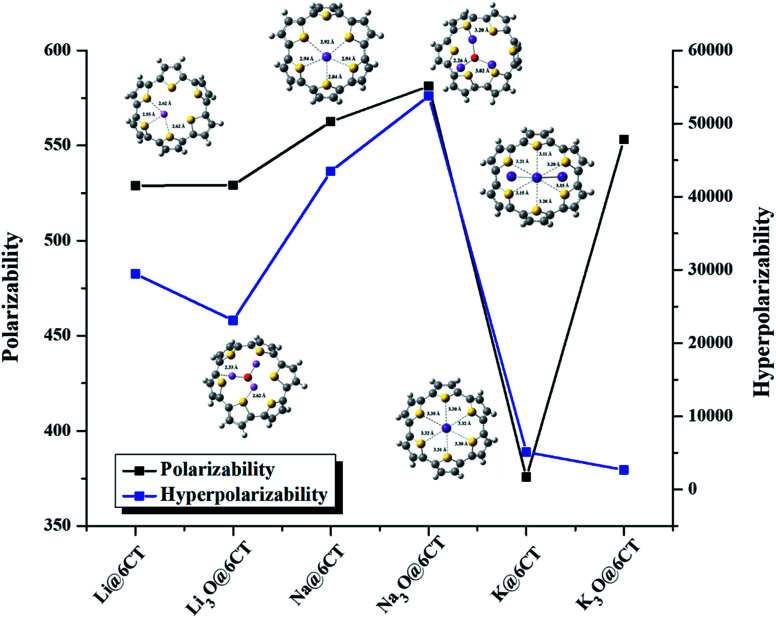
Graphical representation of polarizability and hyperpolarizability of AA@6CT and SA@6CT complexes.

The static hyperpolarizabilities of the designed complexes have been computed to explore their NLO responses and the results are listed in Table 3 & Fig. 5. The static hyperpolarizability (*β*_o_) of isolated 6CT is 2 × 10^4^ au. The complexation of alkali metal and superalkalis remarkably boost the *β*_o_ of 6CT to lie in the range of 2 × 10^4^–5 × 10^4^ au. Surprisingly, the NLO responses of Li and K doped 6CT is higher than their corresponding superalkalis complexes. The lower NLO responses of Li_3_O@6CT and K_3_O@6CT complexes are probably due to the higher vertical ionization energy (*V*_IE_) values^33^ which reflects the removal of electron is difficult from these systems. On the hand, Na_3_O@6CT complex exhibits lower *V*_IE_ (4.94 eV) than corresponding Na@6CT complex, therefore, the *β*_o_ of former is far higher than the later complexes. Furthermore, *β*_o_ results reveal that the Na_3_O@6CT complex is highly NLO active among other designed complexes.

To support these findings, the projection of static hyperpolarizability on the dipole moment vector (*β*_vec_) is also computed. The trend of *β*_vec_ is exactly consistent to the *β*_o_ of complexes which reveals that Na_3_O@6CT complex is highly NLO sensitive while K_3_O@6CT complex shows least sensitivity. These findings are well comparable to the above-mentioned electronic parameters especially HOMO–LUMO gap and NBO charge transfer analysis. The hyperpolarizability of superalkalis doped complex agrees well with the previously reported similar superalkalis doped cyclic conjugated ring systems. In our previous study, we have reported that the *β*_o_ of superalkalis doped cyclic oligofuran systems was appreciably high and ranges from 1 × 10^4^ to 2 × 10^4^ au especially in Li_3_O doped complexes.^27^ In another similar study, the reported *β*_o_ of lithium doped cyclic conjugated ring system was 7.3 × 10^3^ au^33^ and 3 × 10^4^ au.^34^ In this study, we found that the NLO responses of alkali and superalkalis doped 6CT complexes have shown good agreement with the previously reported similar systems.^35,36^

Two level model is performed to interpret the effects of change in dipole moment (Δ*μ*), oscillator strength (*f*_o_) and crucial excited energy (Δ*E*_ex_) on the hyperpolarizability of complexes. The two-level model can be defined by the expression below;^37^*β*_o_ ∝ *f*_o_ × Δ*μ*/Δ*E*_ex_^3^

Based on the above equation, the *β*_o_ increases with increasing values of oscillator strength or the change in dipole moment. On the other side, the value of *β*_o_ is lower if a complex exhibits high crucial excited energy. The results illustrate that the Na_3_O@6CT complex shows the highest *β*_o_ which might be due to the largest value of Δ*μ* (6.19 D). The lowest value of crucial excited energy (1.66 eV) is also contributing to the higher hyperpolarizability of Na_3_O@6CT complex. Similarly, the smallest oscillator strength (*f*_o_) (0.04 au) of K_3_O@6CT complex plays a vital role of exhibiting small NLO response.

The static second hyperpolarizability (*γ*_o_) values are also estimated and the results revealed that *γ*_o_ values have trend similar to that of *β*_o_ but the *γ*_o_ values are significantly larger. The *γ*_o_ values of the designed complexes are in the range of 3.6 × 10^5^ to 5.6 × 10^6^ au. According to previous results,^38^ the giant NLO response in the alkali and superalkalis doped cyclic thiophene may be caused by the excess electrons of doped alkali/superalkalis.

Due to the lack of experimental results, the hyperpolarizability of complexes are also calculated at M05-2X and M06-2X with different basis sets including 6-31G(d,p), 6-31+G(d,p) and 6-311+G(d,p) (Table 4). The trend of hyperpolarizability of AA@6CT and SA@6CT complexes at Minnesota functional is quite comparable with LC-BLYP. Like LC-BLYP, the *β*_o_ of Na_3_O@6CT complex is the highest in all the cases with little exception at 6-31G(d,p) and 6-311+G(d,p) basis sets. The *β*_o_ of Na@6CT and Na_3_O@6CT are 2.3 × 10^4^ and 2.9 × 10^4^ au, respectively at M05-2X/6-31+G(d,p) level of theory, the highest compared to other AA@6CT and SA@6CT complexes. At M06-2X, the *β*_o_ values of Na@6CT and Na_3_O@6CT complexes are 2.2 × 10^4^ and 2.6 × 10^4^ au which is quite consistent with the results of competing M05-2X method. Though the trend is same with LC-BLYP but the values of *β*_o_ for complexes are appreciably lower at Minnesota functionals. Moreover, LC-BLYP is a full range separated functional with 1.00 fraction for nonlocal exchange at asymptotic distance.^39,40^ Therefore, the nonlinear optical results of material are more reliable at LC-BLYP^15^ thus, the dynamic hyperpolarizability has been calculated at widely accepted LC-BLYP.

**Table 4:** Hyperpolarizability (*β*_o_ in au) of SA@6CT and AA@6CT complexes at M052X and M062X methods

Complexes	M052X			M062X		
	6-31G (d,p)	6-31+G (d,p)	6-311+G (d,p)	6-31G (d,p)	6-31+G (d,p)	6-311+G (d,p)
6CT	7.0 × 10^1^	2.1 × 10^2^	2.4 × 10^2^	6.6 × 10^0^	2.3 × 10^2^	2.2 × 10^2^
Li@6CT	1.6 × 10^4^	1.8 × 10^2^	1.8 × 10^4^	1.6 × 10^4^	1.8 × 10^4^	1.8 × 10^4^
Li_3_O@6CT	1.6 × 10^4^	1.8 × 10^4^	1.9 × 10^4^	1.6 × 10^4^	1.9 × 10^4^	1.9 × 10^4^
Na@6CT	2.0 × 10^4^	2.3 × 10^4^	3.5 × 10^3^	1.9 × 10^4^	2.2 × 10^4^	2.1 × 10^4^
Na_3_O@6CT	2.6 × 10^4^	2.9 × 10^4^	3.1 × 10^4^	1.1 × 10^5^	2.6 × 10^4^	2.5 × 10^4^
K@6CT	3.0 × 10^2^	3.8 × 10^2^	4.6 × 10^2^	4.1 × 10^2^	1.9 × 10^2^	2.6 × 10^2^
K_3_O@6CT	6.1 × 10^4^	5.9 × 10^3^	2.5 × 10^3^	4.0 × 10^4^	3.3 × 10^3^	9.7 × 10^3^

### 4.2 Dynamic NLO responses

The dynamic first hyperpolarizability coefficients *i.e.* electro-optic Pockel's effect (EOPE) with *β*(−*ω*, *ω*, 0) and the second-harmonic generation (SHG) with *β*(−2*ω*, *ω*, *ω*) of the designed complexes are calculated and the values are given in Table 5. The frequency dispersion analysis was performed with the wavelengths of 532 nm (0.0856 au) and 1064 nm (0.0428 au) in order to provide a frequency dependent NLO response at the routinely utilized Nd:YAG laser frequencies. The calculated results revealed that the first hyperpolarizability *β*(*ω*) coefficients are dependent on the wavelengths. One can see that the static first hyperpolarizability of the designed complexes are in the range of 3 × 10^3^ to 5 × 10^4^ au while the frequency dependent response are largely enhanced. The EOPE values at 1064 nm are in the range of 3.1 × 10^3^ to 1.9 × 10^6^ au. The EOPE values are further enhanced to the range of 3.6 × 10^4^ to 7.2 × 10^7^ au at shorter wavelength of 532 nm. This indicates that EOPE values are more susceptible to shorter wavelength (532 nm). The second-harmonic generation (SHG) coefficient *β*(−2*ω*, *ω*, *ω*) values of the designed complexes lie in the range of 8.8 × 10^3^ to 2.0 × 10^8^ au at 1064 nm and decreases to the range of 2.5 × 10^4^ to 2.2 × 10^7^ au which indicates that the resonant enhancement for SHG process takes place at higher wavelength (1064 nm). Overall SHG process has stronger NLO response than EOPE at wavelength of 1064 nm for all designed complexes however, shorter wavelength 532 nm induces slightly stronger EOPE response than SHG process for Li_3_O@6CT, Na@6CT, Na_3_O@6CT, and K@6CT complexes.

**Table 5:** Estimated values of *β*(−*ω*, *ω*, 0), *β*(−2*ω*, *ω*, *ω*), *γ*(−*ω*, *ω*, 0, 0), *γ*(−2*ω*, *ω*, *ω*, 0), *γ*^DFWM^, and *n*_2_ (cm^2^ W^−1^) in au

Parameters	Frequency *ω*	Li@6CT	Li_3_O@6CT	Na@6CT	Na_3_O@6CT	K@6CT	K_3_O@6CT
*β*(−*ω*, *ω*, 0)	1064 nm	4.4 × 10^5^	1.7 × 10^5^	9.8 × 10^4^	1.9 × 10^6^	3.1 × 10^3^	5.6 × 10^3^
	532 nm	4.8 × 10^4^	3.6 × 10^4^	2.7 × 10^5^	7.2 × 10^7^	8.2 × 10^4^	8.8 × 10^4^
*β*(−2*ω*, *ω*, *ω*)	1064 nm	7.7 × 10^5^	2.7 × 10^5^	5.6 × 10^5^	2.0 × 10^8^	8.8 × 10^3^	1.6 × 10^5^
	532 nm	9.2 × 10^4^	2.5 × 10^4^	5.8 × 10^4^	2.2 × 10^7^	4.9 × 10^4^	1.7 × 10^5^
*γ*(−*ω*, *ω*, 0,0)	1064 nm	5.1 × 10^7^	7.8 × 10^6^	1.4 × 10^8^	1.5 × 10^9^	8.1 × 10^5^	1.0 × 10^6^
	532 nm	4.0 × 10^6^	4.6 × 10^6^	3.5 × 10^7^	3.7 × 10^10^	2.2 × 10^8^	4.4 × 10^7^
*γ*(−2*ω*, *ω*, *ω*, 0)	1064 nm	2.1 × 10^8^	7.4 × 10^7^	9.4 × 10^7^	1.3 × 10^11^	5.5 × 10^6^	1.4 × 10^7^
	532 nm	1.5 × 10^7^	1.0 × 10^7^	3.2 × 10^6^	2.9 × 10^10^	2.4 × 10^7^	6.7 × 10^8^
*γ* ^DFWM^	1064 nm	1.2 × 10^8^	3.2 × 10^7^	1.7 × 10^8^	4.6 × 10^10^	7.9 × 10^5^	5.7 × 10^6^
	532 nm	8.2 × 10^6^	7.4 × 10^6^	3.5 × 10^7^	4.6 × 10^10^	2.3 × 10^8^	2.7 × 10^8^
*n*_2_ (cm^2^ W^−1^)	1064 nm	1.0 × 10^−14^	2.7 × 10^−15^	1.4 × 10^−14^	3.8 × 10^−12^	6.6 × 10^−17^	4.7 × 10^−16^
	532 nm	6.9 × 10^−16^	6.2 × 10^−16^	2.9 × 10^−15^	3.8 × 10^−12^	1.9 × 10^−14^	2.2 × 10^−14^

The dynamic second hyperpolarizability coefficients including dc-Kerr effect *γ*(−*ω*, *ω*, 0, 0) and the electric field-induced second harmonic generation (ESHG) *γ*(−2*ω*, *ω*, *ω*, 0) are also computed and the results are listed in the Table 5. Like dynamic first hyperpolarizability analysis, the frequency dispersion analysis was performed with the same wavelengths of 532 nm (0.0856 au) and 1064 nm (0.0428 au). From Table 5, one can see that the designed complexes have remarkably large dc-Kerr effect (up to 3.7 × 10^10^ au) and ESHG (up to 1.3 × 10^11^ au). The largest dc-Kerr effect and ESHG response was shown by Na_3_O@6CT complex. From static, dc-Kerr and ESHG second hyperpolarizability coefficients, degenerate four-wave mixing (DFWM), *γ*^DFWM^(*ω*) = *γ*(−*ω*, *ω*, −*ω*, *ω*) was calculated using the following equation:^41^*γ*^DFWM^(−*ω*, *ω*, −*ω*, *ω*) ≈ (1/3)*γ*(−2*ω*, *ω*, *ω*, 0) + *γ*(−*ω*, *ω*, 0, 0) − (1/3)*γ*(0, 0, 0, 0)

Then from DFWM values we can evaluate the quadratic nonlinear refractive index (*n*_2_) using the following equation:^42^*n*_2_ (cm^2^ W^−1^) = 8.28 × 10^−23^*γ*^DFWM^ (au)

The values of quadratic nonlinear refractive index (*n*_2_) are significantly large for all complexes. At wavelength of 1064 nm, the *n*_2_ values ranges from 6.6 × 10^−17^ to 3.8 × 10^−12^ au and at 532 nm, the *n*_2_ values are in the range of 6.9 × 10^−16^ to 3.8 × 10^−12^ au. The *n*_2_ values as a function of wavelength confirm that the amplitude of the second order nonlinear optical response is expected to vary with changes in wavelength as well as different alkali metal.

## 5 Conclusions

In this research, a series of complexes have been designed by doping alkali metal atoms (Li, Na and K) and superalkalis (Li_3_O, Na_3_O & K_3_O) for nonlinear optical applications. The interaction energies at M05-2X and M06-2X with 6-31G(d,p), 6-31+G(d,p) and 6-311+G(d,p) basis sets reveal that the reported complexes are thermodynamically stable. In comparison, the interaction stabilities of superalkalis@6CT complexes are far higher than alkali atoms@6CT complexes. Furthermore, the doping of alkali metals and their corresponding superalkalis leads to remarkably high NLO responses. Among all the complexes, Na_3_O@6CT complex exhibits highest NLO response. The static hyperpolarizability of Na_3_O@6CT complex is 5 × 10^4^ au along with pronounced *β*_vec_ value (2.5 × 10^4^ au) and significantly large quadratic nonlinear optical response (3.8 × 10^−12^ au). The electronic properties provide evidence of high NLO response of Na_3_O@6CT complex. For example, Na_3_O@6CT complex shows an appreciable amount of NBO charge transfer (1.53*e*^−^) along with the considerably low *E*_H–L_ gap (2.96 eV). The absorption spectrum also illustrates the ultra-high transparency of Na_3_O@6CT complex in UV-vis region. Finally, the improved second-harmonic generation, electro-optic Pockel's effect and nonlinear refractive index of our alkali/superalkali doped 6CT complexes open new prospective for the designing of new NLO material, especially Na_3_O@6CT, for opto-electronic applications.

## Conflicts of interest

There are no conflicts to declare.

## Supplementary Material

RA-011-D0RA08099C-s001
