# Supplementary material for: Remarkable static and dynamic NLO response of alkali and superalkali doped macrocyclic [hexa-]thiophene complexes; a DFT approach
Source: RSC Adv. 2021 Jan 20;11(7):4118–28. doi: 10.1039/d0ra08099c (PMC8694385; doi:10.1039/d0ra08099c)
Supplement: RA-011-D0RA08099C-s001 [file RA-011-D0RA08099C-s001.pdf]

Electronic Supplementary Material (ESI) for RSC Advances.
This journal is © The Royal Society of Chemistry 2021

Remarkable static and dynamic NLO response of alkalis and superalkalis doped macrocyclic [hexa-]thiophene complexes; a DFT approach

Hasnain Sajid, Faizan Ullah, Sidra Khan, Khurshid Ayub, Tariq Mahmood*

Department of Chemistry, COMSATS University Islamabad, Abbottabad Campus, Abbottabad-22060, Pakistan

*To whom correspondence can be addressed: E-mail: mahmood@cuiatd.edu.pk (T. M)

Li@6CT

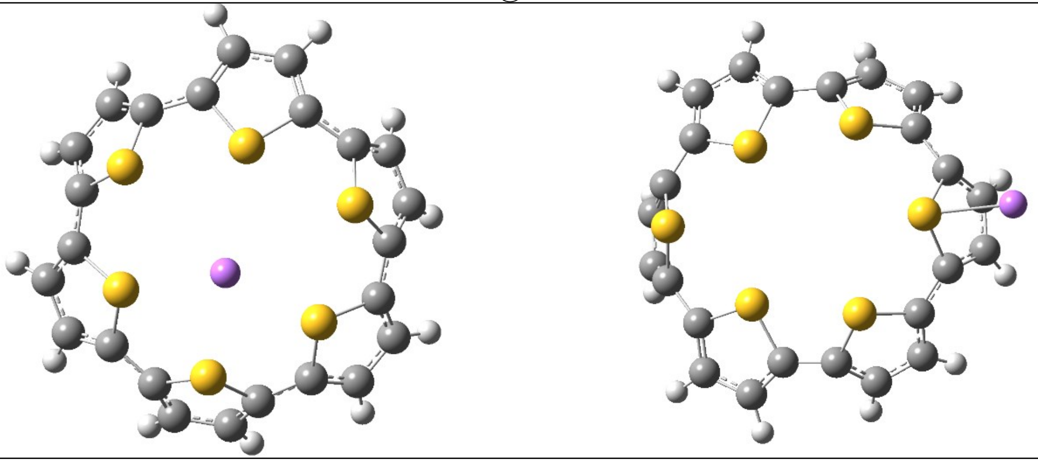

Na@6CT

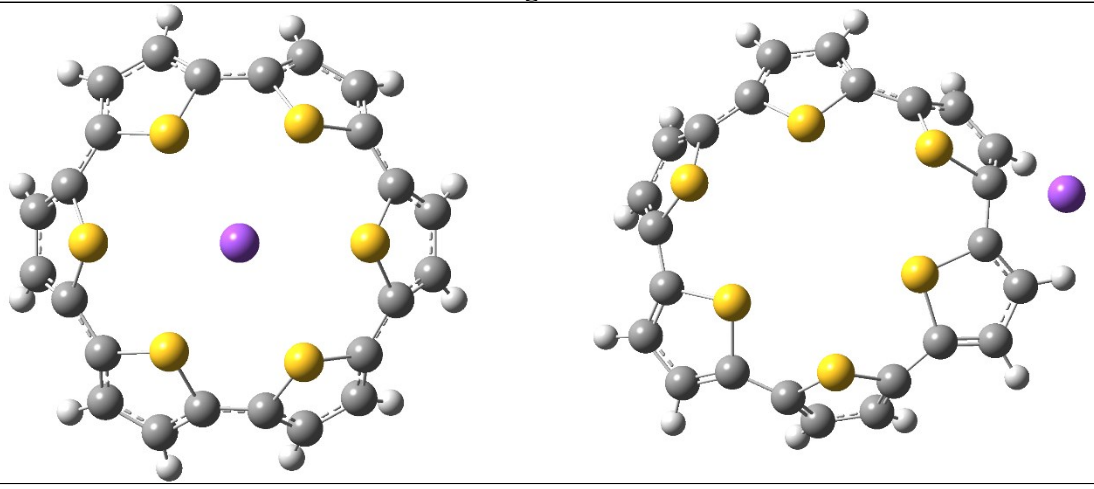

K@6CT

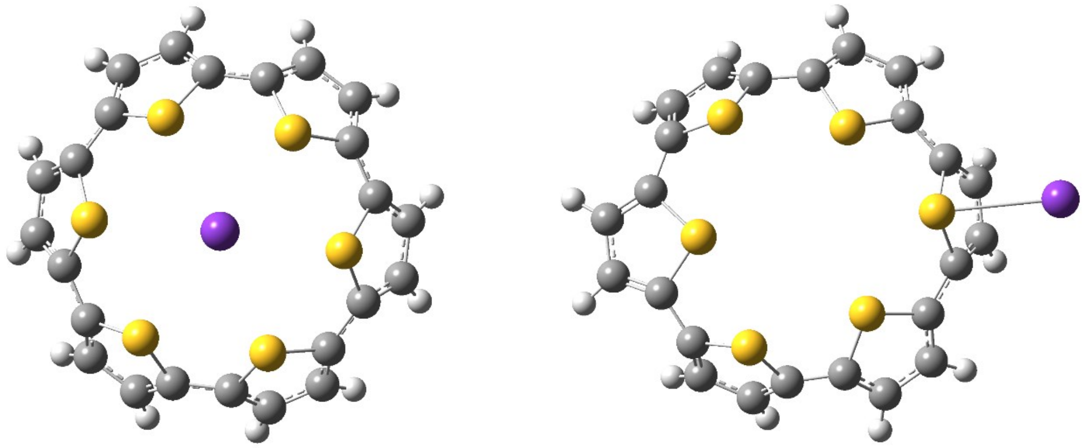

Figure. S1. The possible optimized geometries of AA@6CT complexes.

Li_3O@6CT

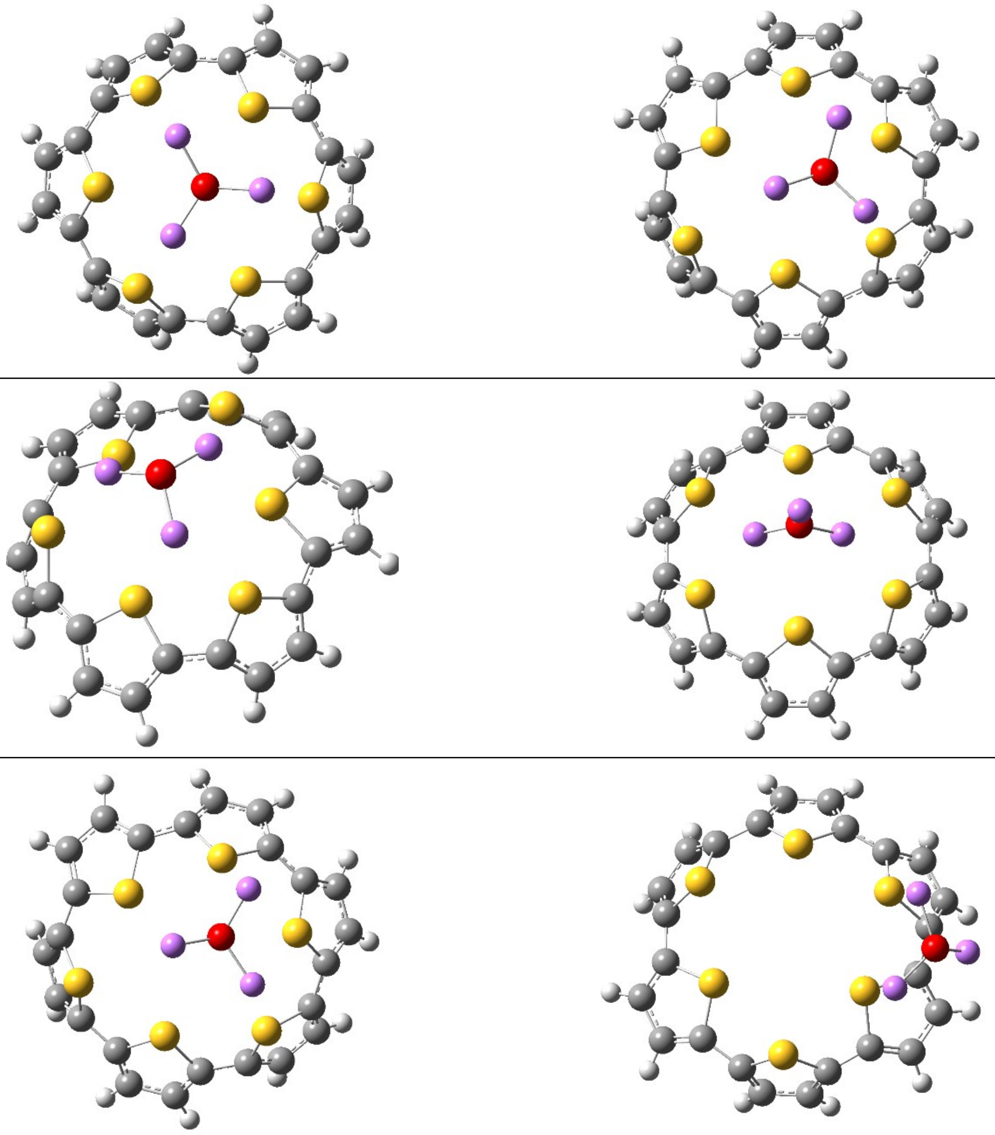

Figure. S2. The possible optimized geometries of Li_3O@6CT complexes.

Na_3O@6CT

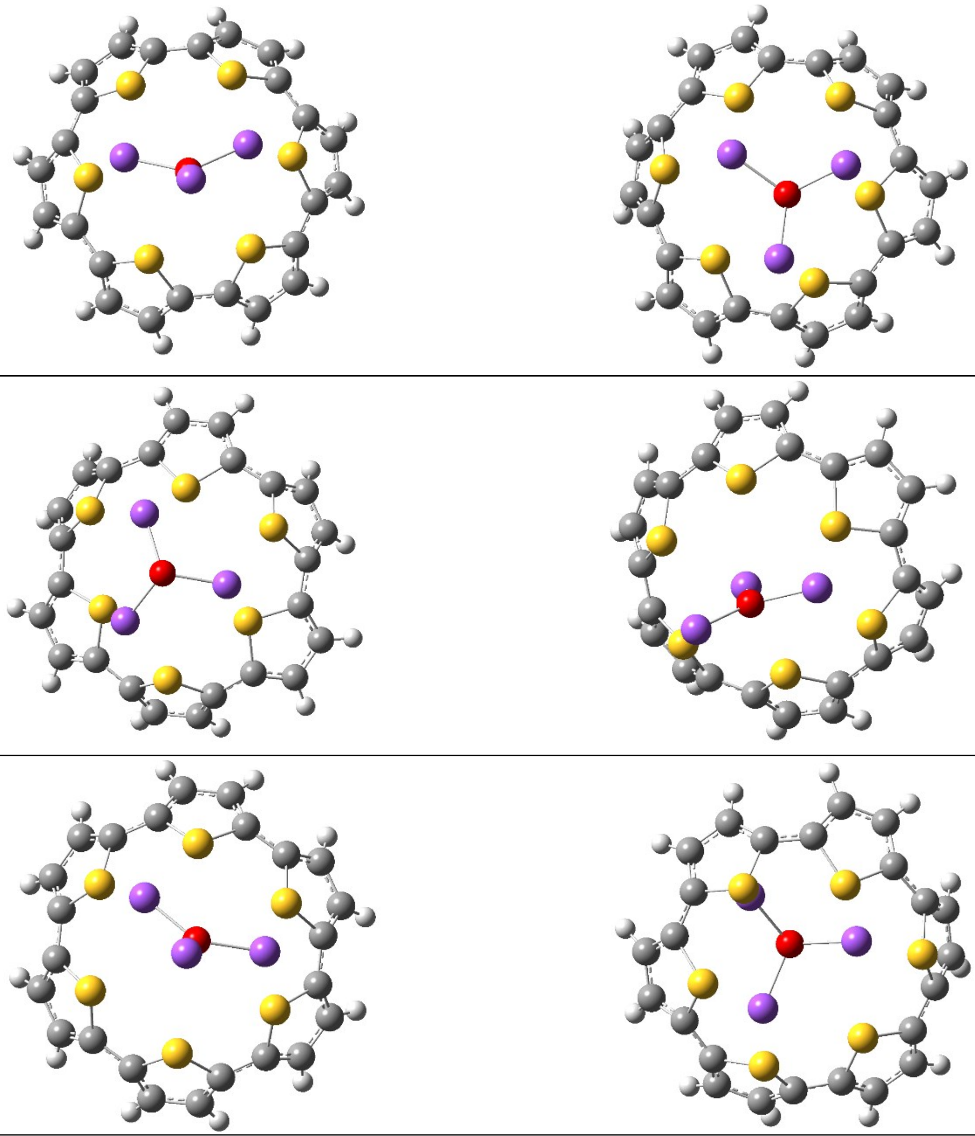

Figure. S3. The possible optimized geometries of Na_3O@6CT complexes.

$K_3O@6CT$

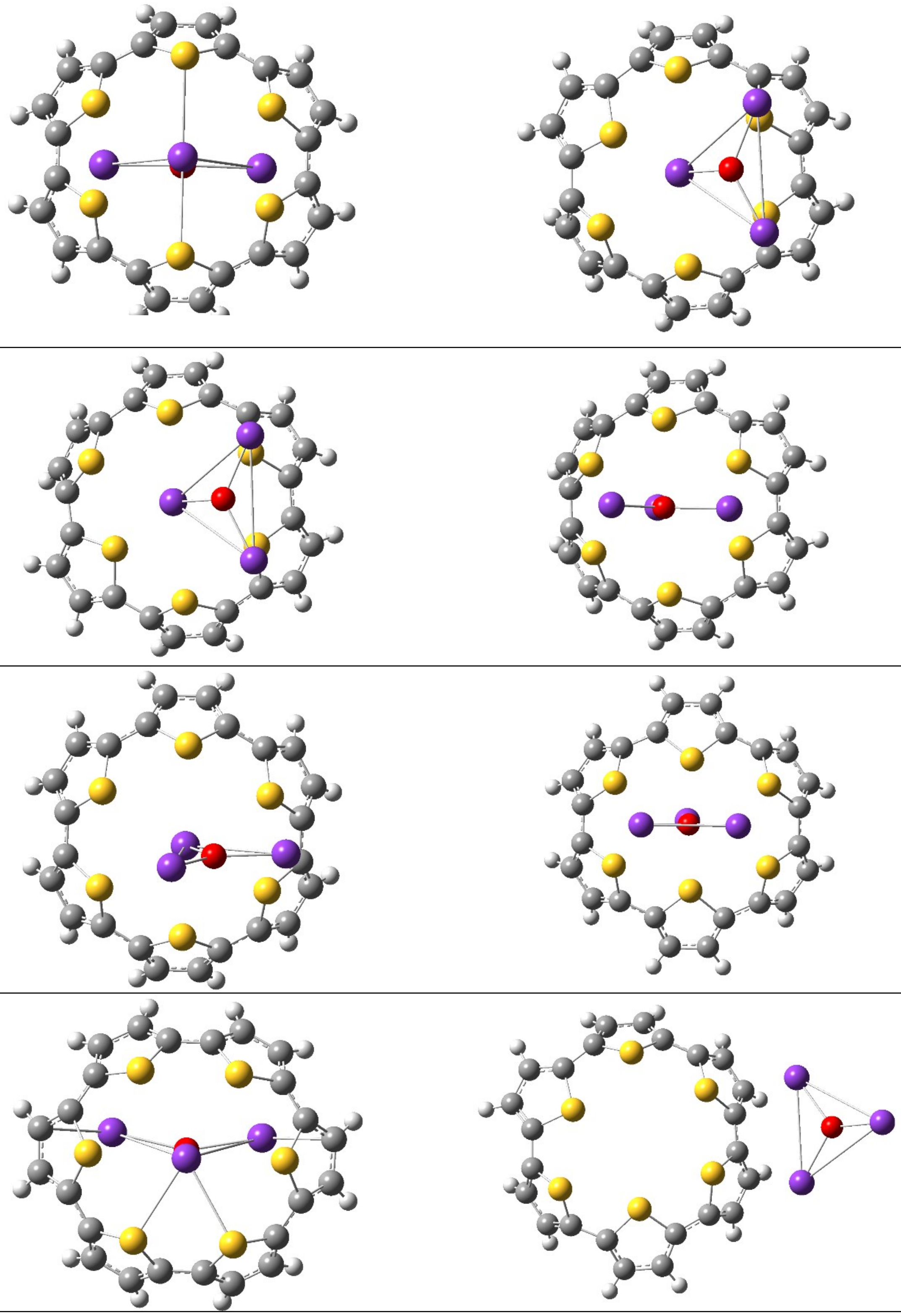

Figure. S2. The possible optimized geometries of $K_3O@6CT$ complexes.
